# Supplementary material for: Patterns of Intron Gain and Loss in Fungi
Source: PLoS Biol. 2004 Nov 30;2(12):e422. doi: 10.1371/journal.pbio.0020422 (PMC532390; doi:10.1371/journal.pbio.0020422)
Supplement: Table S1 — Also available at http://genes.mit.edu/NielsenEtAl/. (4.3 MB ZIP). [file pbio.0020422.st001.zip › NielsenEtAl/html/36.html]

AN5122.1.NCU01550.1.MG01058.1.FG10737.1


```
 CLUSTAL W (1.82) Multiple Sequence Alignments - Introns Inserted


Sequence 1: MG01058.1	276 aa
Sequence 2: FG10737.1	258 aa
Sequence 3: NCU01550.1	278 aa
Sequence 4: AN5122.1	259 aa
Alignment Length: 280 aa
Number Identitical Residues: 155 aa
Alignment Score (without introns) 7020


MG01058.1 	MGAMEDQLQR-LGSVLDSLDARVKKLEQHPTLG-GGSSTAQEIRMILIGPPGAG1KGTQA
NCU01550.1	MGFIDDEVKR-LGDVIASLEDRVRSLETRDFSG-KPVTTAEQLRMVLMGPPGAG~KGTQA
FG10737.1 	MGFIEDELKQ-LKDVISTIDTRIKKLEARAT---GGPVSTDEIRMILIGPPGAG1KGTQA
AN5122.1  	MAPITDDVVAGLKSTIGKLEARIEDLESRLGGEPKPKSIAEHMRIILMGPPGAG1KGTQA
          	*. : *::  .* ..: .:: *:..** :    .     ::.:*::*:****** *****

MG01058.1 	PKIKEKFSCCHL0ATGDMLRSQVAKKTPLGREAKKIMDQGGLVSDDIVIGMIKEELDTNV
NCU01550.1	PKIKEKFNCCHL0ATGDMLRAQVAKGTALGKQAKKIMNEGGLVSDDIVIGMIKDELENNK
FG10737.1 	PKIKERFSCCHL0ATGDMLRSQVTKKTPLGVEAKKIMDAGGLVSDEIMIGMIKEELNNNK
AN5122.1  	PKIKEKYCVCHL0ATGDMLRSQVAKKTDLGREAKKIMDQGGLVSDEIMVNMIKSELENNA
          	*****::  *** *******:**:* * ** :*****: ******:*::.***.**:.* 

MG01058.1 	ECKGG2FILDGFPRTVPQAQSLDAMLQARNQKLQHAVELQIDDALLVARITGRLVHPASG
NCU01550.1	ECQGG2FILDGFPRTVPQAEGLDAMLRERNLPLQHAVELKIDDSLLVARITGRLVHPASG
FG10737.1 	ECQGG2FILDGFPRTVPQAEGLDAMLAERKQKLQHAVELQIDDSLLVARITGRLVHPASG
AN5122.1  	ECKNG2FILDGFPRTVAQAERLDEMLVARNQKLQHAIELKIDDALLVARITGRLVHPASG
          	**:.* **********.**: ** **  *:  ****:**:***:****************

MG01058.1 	RSYHTTFNPPKKAMTDDVTGEPLIQRSDDNADALKKRLATYHSQTAPVVDYYRKTGIWKP
NCU01550.1	RSYHRIFNPPKDDMKDDITGEPLVQRSDDNAEALRKRLETYHKQTAPVVGYYQNTGIWKA
FG10737.1 	RSYHTTFNPPKEYMKDDITGEPLIQRSDDNADALKKRLVTYHKQTKPVVGYYQKTGIWSG
AN5122.1  	RSYHKIFNPPKEAMKDDITGEPLVQRSDDNAEALKKRLVTYHAQTAPVCDYYKKTGIWRG
          	****  *****. *.**:*****:*******:**:*** *** ** ** .**::****  

MG01058.1 	IDASQEPGTVWKSLLNIFDGDAKKASSAGGGILDKLAASGR--
NCU01550.1	IDASQEPAQVWKSLLAIFEGDKAKASSAGSGIMSKIASAAKSS
FG10737.1 	LDASQEPGQVWKNMLKVLN--EKRGQN----------------
AN5122.1  	IDASQEPGQVWKSLLGVFN---NKN------------------
          	:******. ***.:* :::    :
```
